# Enterotoxigenic Escherichia coli in Blantyre, Malawi

**DOI:** 10.1099/acmi.0.000885.v3

**Published:** 2024-11-18

**Authors:** Philip M. Ashton, Zefaniah Joel Katuah, Arnold Botomani, Belson M. Kutambe, Nigel A. Cunliffe, Astrid von Mentzer, Chisomo Msefula, Khuzwayo C. Jere

**Affiliations:** 1 Malawi Liverpool Wellcome Programme, Blantyre, Malawi; 2 Institute of Infection, Veterinary, and Ecological Sciences, University of Liverpool, Liverpool, UK; 3 Kamuzu University of Health Sciences, Blantyre, Malawi; 4 Department of Microbiology and Immunology, Institute of Biomedicine, Sahlgrenska Academy, University of Gothenburg, Gothenburg, Sweden

**Keywords:** diarrhoea, *E. coli*, genomics

## Abstract

We announce the deposition of the first two enterotoxigenic *Escherichia coli* (ETEC) genomes from Malawi. They were isolated from the faeces of asymptomatically infected children obtained in 2014. Both genomes encode the porcine variant of the heat-labile toxin and no known ETEC colonization factors.

## Data Summary

Genome assemblies are available from the NCBI with the accessions GCA_040576965.1 for BND1DE11 and GCA_040712285.1 for BND1D31.

## Introduction

Childhood diarrhoea is one of the most pressing problems in global health, causing the deaths of around 446,000 children under the age of 5 years in 2016 globally [1]. Enterotoxigenic *Escherichia coli* (ETEC) is a significant cause of diarrhoea in many low- and middle-income countries, causing 75 million diarrhoeal episodes in children under 5 years(1). In Malawi, 31.1% of children hospitalized at Queen Elizabeth Central Hospital with diarrhoea between 2012 and 2015 were infected with ETEC, and it had the highest attributable fraction of any bacterial diarrhoeal pathogen (12.7%) [2].

The World Health Organization has published preferred product characteristics for vaccines against ETEC (https://cdn.who.int/media/docs/default-source/immunization/final-etec-ppc-2021eng.pdf). However, ETEC is a complex pathogen, with disease caused by at least 21 different lineages, each of which draws from a pool of 29 antigenically distinct colonization factors (adhesion factors enabling the adherence of the pathogen to the human gut), and two enterotoxins with additional subgroups [3]. There are various ETEC vaccines in development [4], which cover only a fraction of the diversity of colonization factor antigens. Therefore, it is important to know which colonization factors are present in high-burden settings such as Malawi.

## Description of dataset

To contribute to this understanding, we isolated ETEC from archived samples from a diarrhoea surveillance study [5], using MacConkey agar for culture from faeces and toxin-PCR (LT, STh and STp) to identify ETEC colonies [6]. DNA was extracted using the Qiasymphony robot using the DSP Virus/Pathogen Kit (Qiagen, UK). We prepared DNA libraries for sequencing using the rapid barcoding kit (SQK-RBK004) and carried out genome sequencing using the MinION and an R.9.4.1 flow cell (Oxford Nanopore Technologies, UK). Library prep and sequencing were carried out for two ETEC isolates (BND1D3-1 and BND1DE-11) obtained from asymptomatically infected children in 2014. After basecalling with guppy v5.0.7 [7], the reads were *de novo* assembled with flye v.2.9 with the parameter ‘—nano-raw’ [8]. No read trimming was carried out. blast analysis was carried out against a database of ETEC virulence factors, including LT, ST and representatives of colonization factors (https://github.com/avonm/ETEC_vir_db). Genome assemblies are available from NCBI with the accessions GCA_040576965.1 for BND1DE11 and GCA_040712285.1 for BND1D31.

Overall genome assembly characteristics for BND1D31 were a chromosome of 5.1 Mbp, three putative plasmids of 109 Kbp, 20 Kbp and 10 Kbp. NCBI PGAP (Prokaryotic Genome Annotation Pipeline) annotation pipeline identified 4049 protein-coding genes out of a total of 5228 [9]. The BND1DE11 genome assembly had four contigs that were predicted to belong to the chromosome of 4.7 Mbp, 432 Kbp, 30 Kbp and 14 Kbp and three putative plasmids of 111 Kbp, 20 Kbp and 15 Kbp, respectively. There were a total of 5307 genes predicted, 4155 of which were protein coding. The low proportion of protein-coding genes is likely due to frameshift errors that are common in Oxford Nanopore Technologies sequencing.

**Fig. 1.**
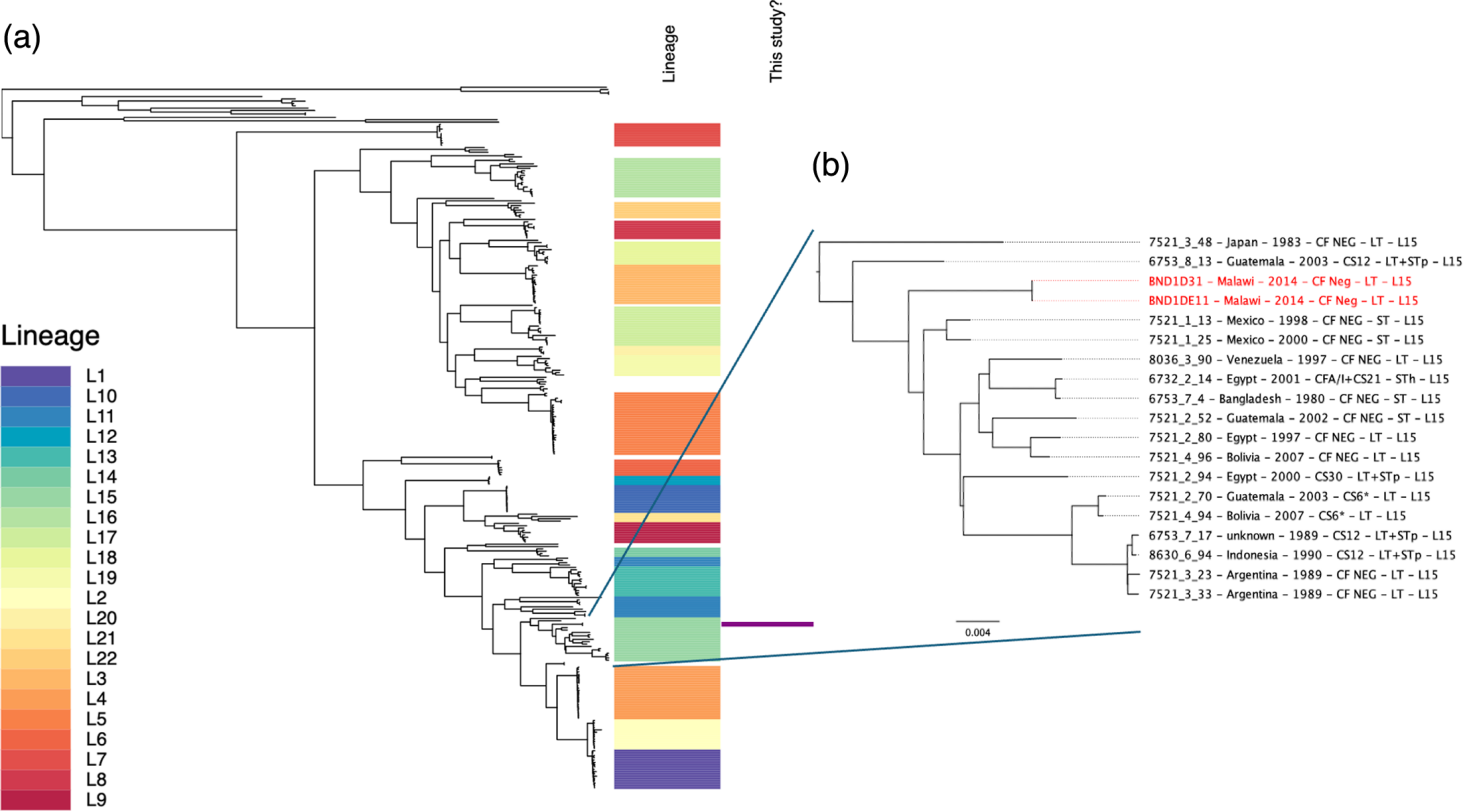
(a) A maximum likelihood phylogenetic tree of 301 ETEC genomes from von Mentzer *et al.* [3] and the 2 Malawian genomes isolated as part of this study. The first column is the lineage of each genome, the second colony indicates the genomes generated by this study. (b) An inset focussing on L15 of the ETEC phylogeny, which contains the genomes generated as part of this study (highlighted in red).

When the ETEC genomes were compared against a virulence factor database using blast, the most similar ETEC toxin sequence identified in both samples was the ‘porcine’ subtype of the heat-labile toxin subunits A and B (Table 1). No other known ETEC colonization factor genes were identified. We placed the two genomes into the context of 301 geographically and temporally diverse ETEC [3] using SKA to align genomes [10] and IQ-TREE using the model GTR+ASC+G4 [11] (Fig. 1). This showed that the Malawian genomes belonged to Lineage 15, a globally distributed lineage found in South and Central America, Asia and North Africa. This is the first report of this lineage from sub-Saharan Africa. Although the nearest phylogenetic neighbours of our Malawian genomes are quite distant genomes from Mexico, it is notable that they are also colonization factor negative and encode LTp.

**Table 1.** Results of blast comparison of ETEC genomes generated as part of this study against a database of virulence factors

Sample	Contig	Subject sequence ID	Identity (%)	Length	Coverage	Mismatch	Gap open	Query start	Query end	Subject start	Subject end	E value	Bit score
BND1D3-1	contig_2	31919_4–192_05220_eltA_LTp_heat-labile_toxin_subunit_A	100	657	100	0	0	15,404	16,060	1	657	0	1214
BND1D3-1	contig_2	31919_4–192_05221_eltA_LTp_heat-labile_toxin_subunit_B	100	375	100	0	0	16,057	16,431	1	375	0	693
BND1D3-1	contig_2	31919_4–192_05214_M94_stb_STb_heat-stabile_toxin_subunit_b	100	216	100	0	0	10,494	10,709	216	1	8.66E-111	399
BND1DE-11	contig_5	31919_4–192_05220_eltA_LTp_heat-labile_toxin_subunit_A	100	657	100	0	0	17,139	17,795	1	657	0	1214
BND1DE-11	contig_5	31919_4–192_05221_eltA_LTp_heat-labile_toxin_subunit_B	100	375	100	0	0	17,792	18,166	1	375	0	693
BND1DE-11	contig_5	31919_4–192_05214_M94_stb_STb_heat-stabile_toxin_subunit_b	99.537	216	100	0	1	12,229	12,443	216	1	1.47E-108	392

## Discussion

This work presents the first ETEC genomes from Malawi, indicating that they encode no known colonization factors. Further analysis of these genomes should include searches for putative novel colonization factors. More work is required to understand the population in greater depth and investigate the possibility of previously uncharacterized virulence factors, in particular from participants with moderate-to-severe diarrhoea.

## Abbreviations

ETEC: enterotoxigenic *Escherichia coli*
